# Facilitators and barriers to generic and biosimilar medications in the Middle East and North Africa: insights from physicians and pharmacists—a systematic review

**DOI:** 10.1007/s00228-025-03819-5

**Published:** 2025-03-14

**Authors:** Kefah Ali Alqawasmeh, Thomas Mason, Abigail Morris, Wael Hafez, Thekra Hasan, Sondos Taher, Rania Al Dweik

**Affiliations:** 1 The Division of Health Research, Lancaster University, Lancaster, UK; 2 Medical Research and Clinical Studies Institute, The National Research Centre, Cairo, Egypt; 3 NMC Royal Hospital, Khalifa City, Abu Dhabi, United Arab Emirates; 4 Department of Health Abu Dhabi, Abu Dhabi, United Arab Emirates; 5 Department of Public Health, College of Health Sciences, Abu Dhabi University, Abu Dhabi, United Arab Emirates; 6 Department of Epidemiology and Population Health, American University of Beirut, Beirut, Lebanon

**Keywords:** Generics and biosimilar drugs, Middle East healthcare systems, Facilitators, Barriers, Physicians

## Abstract

**Background:**

The adoption of generic and biosimilar medications is crucial for improving healthcare accessibility and cost savings in the Middle East and North Africa (MENA) region. Understanding the factors that influence their acceptance is crucial for developing effective strategies for promoting their use.

**Purpose:**

This systematic review aimed to examine the facilitators and barriers identified by healthcare professionals while prescribing and dispensing generic and biosimilar medications in the MENA region, focusing on their perceptions, knowledge, and attitudes.

**Methods:**

Following Cochrane guidelines and the “Guidance on the Conduct of Narrative Synthesis in Systematic Reviews,” a comprehensive search of electronic databases and grey literature was conducted from 2012 to 2024. Studies assessing physicians’ and pharmacists’ perspectives on generics and biosimilars in the MENA region were included. Quality appraisal was performed using a standardized tool, the mixed methods appraisal tool (MMAT). The findings were synthesized using a descriptive analysis.

**Results:**

Of the 3570 screened citations, 39 met the inclusion criteria. Among them, 25 studies addressed facilitators and barriers to adopting generic medications, whereas 15 focused on biosimilars. Facilitators of generic medications included understanding the use of generics as cost-effective substitutes, supportive government policies, generic medication awareness, and pharmacists’ empowerment to substitute medications. Barriers included knowledge gaps leading to distrust in efficacy and safety, the influence of pharmaceutical companies, cultural biases favoring brand name drugs, regulatory challenges, low consumer awareness, and concerns about pharmacists’ profitability. Facilitators for biosimilars were mostly similar to generics, with an added emphasis on access benefit recognition when using biosimilars. Unique barriers included concerns about the lack of long-term safety data, hesitancy toward non-medical switching, and nocebo effect concerns. The quality assessment indicated that most studies were of moderate quality, with limitations such as sample size and representativeness, validity of the measurement tools, and potential biases of the researchers.

**Conclusion:**

Significant knowledge gaps regarding regulatory approval, safety, and efficacy hinder the adoption of generic drugs and biosimilars in MENA. Targeted educational initiatives at the regulatory and payer levels are essential for bridging these gaps, enhancing awareness, and fostering acceptance. Implementing comprehensive educational programs for physicians and pharmacists is crucial to support the transition toward the greater use of generics and biosimilars.

**Supplementary Information:**

The online version contains supplementary material available at 10.1007/s00228-025-03819-5.

## Introduction

The adoption and uptake of generics and biosimilars in the Middle East and North Africa (MENA) region remains suboptimal despite their potential to reduce healthcare costs and improve access to essential medications. Several studies have indicated that physicians and pharmacists exhibit varying levels of acceptance, with pharmacists generally more supportive of generic substitution, whereas physicians demonstrate higher skepticism toward both generics and biosimilars [1, 2]. Key barriers include knowledge gaps, regulatory inconsistencies, and cultural resistance, all of which contribute to delayed adoption [3, 4]*.* Although some MENA countries have introduced policies to encourage generic and biosimilar use, these measures are often inconsistently implemented and lack harmonization across the region [5, 6].

By contrast, Europe and the USA have implemented successful policy-driven strategies to accelerate the uptake of generics and biosimilars. In the European Union (EU), biosimilars saved approximately €50 billion between 2016 and 2021 owing to competitive pricing policies [7]. The USA projects biosimilar-driven savings of $25 billion to $100 billion over a 10-year period [8–10], supported by robust regulatory frameworks that encourage biosimilar adoption through interchangeability designations and substitution laws [11]*.* In comparison, the MENA region lacks a unified strategy for biosimilars and generics, with some countries implementing price control measures, while others rely on prescriber autonomy to influence uptake [12–14].

Several MENA countries have introduced policies to promote generic and biosimilar use. However, inconsistent implementation remains a major challenge. Saudi Arabia mandates that generics be priced at least 30% lower than branded drugs, while its National Transformation Program (NTP) 2030 seeks to increase its use [15]. Jordan has adopted bioequivalence guidelines aligned with the FDA and EMA**,** and pharmacists are still restricted from substituting generic without physician approval [16, 17]. Egypt’s Unified Procurement Authority (UPA) prioritizes local generic production, but physician reluctance due to trust issues with domestic pharmaceuticals remains a barrier(14, 18)*.* Similarly, Lebanon and the UAE have WHO-aligned regulatory frameworks, but enforcement is weak, leading to branded drugs dominating the market [13, 19]*.* Despite these efforts, regulatory inconsistencies, prescription resistance, and low public awareness continue to hinder the adoption of cost-saving alternatives [20, 21].

Regulatory agencies such as the World Health Organization (WHO), US Food and Drug Administration (FDA), and European Medicines Agency (EMA) have established clear definitions and approval pathways for generics and biosimilars. According to the WHO, generic drugs are pharmaceutical products designed to be interchangeable with an innovator product**,** manufactured without a license from the originator company**,** and introduced after patent expiration [22]. Regulatory agencies require bioequivalence studies to ensure that generics exhibit comparable pharmacokinetics and pharmacodynamics to their reference products [23]**.**

However, biosimilars differ significantly from generics because of their biological origins and complex approval processes. The WHO defines biosimilars as biotherapeutic products that closely resemble a licensed reference product in terms of quality, safety, and efficacy [24] Unlike generics, biosimilars are not identical to their reference products because of inherent variability in biological manufacturing. The FDA requires biosimilars to demonstrate high similarity with no clinically meaningful differences, whereas EMA mandates extensive structural, biological, and clinical comparisons to confirm biosimilarity [23]***.*** This distinction leads to longer approval times and stricter post-market monitoring requirements for biosimilars than generics.

Despite these regulatory advancements, many physicians and pharmacists remain unaware of generics, biosimilars, and their rigorous approval processes, which hinders their acceptance by healthcare providers and patients [25]. Physicians express concerns over efficacy, safety, and brand reputation, whereas pharmacists highlight the need for clearer substitution guidelines [21, 26]. These perception-driven barriers, combined with inconsistent regulatory frameworks, slow down the integration of cost-effective alternatives into MENA healthcare systems.

The MENA region accommodates approximately 19 countries, accounting for almost 6% of the world’s population. The region is pencilled as an economic necessity for the globe, generating 60% and 45% of the world’s oil and natural gas supply, respectively [27]. The region encompasses culturally and economically linked countries in the Middle East and North Africa that face growing common healthcare challenges owing to an aging population, rapid population growth, and increasing chronic non-communicable diseases [28, 29]. These factors make it one of the most promising emerging pharmaceutical markets in the world.

Despite the availability of generic and biosimilars, regulatory inconsistencies, cultural preferences, and economic factors have hindered their widespread use. This study systematically reviewed the facilitators and barriers identified by healthcare professionals while prescribing and dispensing generic and biosimilar medications in the MENA region, focusing on their perceptions, knowledge, and attitudes.

## Methods

A systematic review, guided by the “Cochrane Handbook” [30] and “Guidance on the Conduct of Narrative Synthesis in Systematic Reviews” [31] was conducted and reported in accordance with the Preferred Reporting Items for Systematic Reviews and Meta‐Analyses (PRISMA) criteria [32] Our systematic review was registered with the International Prospective Register of Systematic Reviews (PROSPERO) on February 1, 2024, and was last updated on July 21, 2024 (registration number CRD42024508748).

### Search protocol

The search strategies were designed in consultation with a health sciences librarian (L.S.). Literature search strategies relied on Medical Subject Headings (MeSH) and text terminology related to physicians’ and pharmacists’ attitudes toward generics and biosimilars. The following databases were searched for English language restrictions: MEDLINE and EMBASE (via the Ovid interface), CINAHL, PsycINFO (via the EBSCO interface), PubMed, Cochrane Database of Systematic Reviews, Scopus, NHS Economic Evaluation Database, WHO Global Index Medicus, and Grey Literature via Lens. We searched the PROSPERO registry for ongoing and recently completed systematic reviews.

The search terms used (Supplementary Fig. 1) included pharmacists, physicians, general practitioners, medical practitioners, clinicians, healthcare professionals, doctors, generics, biosimilars, advantages, benefits, challenges, barriers, perceptions, attitudes, facilitators, opinions, views, behaviors, and perspectives. The reference lists of eligible studies were also scanned. Citations published between 2012 and 27 February, 2024, were searched to provide updates on previous reviews on generics (Toverud et al.) and biosimilars (Cooper et al.) [33, 34]. Studies published before 2012 were excluded to ensure that the most recent insights were used, as earlier studies may not represent the current dynamics and challenges in the healthcare environment. Moreover, the approach aimed to enhance the relevance and practicality of formulating recommendations based on the latest available evidence.

The majority of studies utilized quantitative cross-sectional designs, with self**-**administered or Web-based questionnaires being the most common data collection methods. Only a few studies have employed qualitative interviews or mixed-method approaches, indicating that most research has focused on survey-based assessments rather than in-depth qualitative analyses.

### Selection process

Inclusion and exclusion criteria were defined according to the Patient, Intervention, Comparator, Outcome, Study Design (PICOS) framework [35]. Only English-language citations were included (Supplementary Table 1). Studies were selected if conducted in MENA countries listed by the World Bank (Algeria, Bahrain, Djibouti, Egypt, Iran, Iraq, Jordan, Kuwait, Lebanon, Libya, Malta, Morocco, Oman, Qatar, Saudi Arabia, Syria, Tunisia, UAE, Palestinian Territories, and Yemen) [36]. Turkey was included to complement the regional use of generic drugs and biosimilars. Only studies addressing pharmacists and physicians were selected, focusing on stakeholders who actively influence the prescribing and dispensing of these products.

After study identification, all citations were imported into Covidence (a Web-based systematic review portal) and duplicates were removed. Two independent reviewers (K.Q. and S.E.) conducted screening at two levels: level 1 screening, using citation title and abstract, was voted as “irrelevant” by both reviewers if the study did not meet the inclusion criteria (see Table 1). A third independent reviewer (R.D.) resolved the conflicts at this stage. Level 2 screening used the full text to determine eligibility and was also performed by reviewers (K.Q. and S.E.); no conflicts were identified at this level.

**Table 1:** Characteristics of included studies in chronological order (*n* = 39)

Authors, date	Country	Generics or biosimilars	Population	Sample size	Design	Data collection	Quality rating
Abduelkarem 2019 [13]	United Arab Emirates	Generics	Physician and patient	100	Quant cross-sectional study	Self-administered questionnaire	High
Abdulah 2019 [38]	Iraq	Generics	Physician	77	Quant cross-sectional study	Self-administered questionnaire	Medium
Abusara 2023 [39]	Jordan	Biosimilars	Pharmacist	400	Quant cross-sectional study	Web‐based questionnaire	Medium
Alahmari 2021[40]	Saudi Arabia	Biosimilars	Pharmacist	319	Quant cross-sectional study	Self-administered questionnaire	Medium
Albadr 2015 [41]	Saudi Arabia	Generics	Pharmacist	20	Qualitative interview	Structured interview	Low
Alkhuzaee 2016 [42]	Saudi Arab	Generics	Pharmacist	121	Quant cross-sectional study	Self-administered questionnaire	High
Almadany 2022 [43]	Saudi Arabia	Biosimilars	Physician	143	Quant cross-sectional study	Web‐based questionnaire	High
Almalki 2020 [44]	Saudi Arabia	Biosimilars	Physician	105	Quant cross-sectional study	Self-administered questionnaire	Medium
Almalki 2020 [45]	Saudi Arabia	Biosimilars	Pharmacist	319	Quant cross-sectional study	Self-administered questionnaire	Medium
Al-saadi 2023 [46]	Oman	Generics	Pharmacist	53	Quant cross-sectional study	Self-administered questionnaire	Medium
Alsufyani 2023 [47]	Saudi Arabia	Generics	Pharmacist	57	Qual interview	structured face-to-face interviews	High
Awada 2023 [48]	Lebanon	Generics and biosimilars	Pharmacist	75	Quant cross-sectional study	Self-administered questionnaire	High
Awaisu 2014 [49]	Qatar	Generics	Pharmacist	118	Quant cross-sectional study	Self-questionnaire	High
Baazaoui 2023 [50]	Tunisia	Generics	Physician	24	Qual interview	in-depth interviews	High
Chahine 2023 [51]	Lebanon	Biosimilars	Pharmacist	354	Quant cross-sectional study	Self-administered questionnaire	High
Demirkan 2022 [52]	Turkey/ USA	Biosimilars	Physician	114	Quant cross-sectional study	Web‐based questionnaire	Medium
El-dahiyat 2013 [53]	Jordan	Generics	Pharmacist	294	Quant cross-sectional study	Self-administered questionnaire	High
El-dahiyat 2014 [54]	Jordan	Generics	Physician	376	Quant cross-sectional study	Self-administered questionnaire	High
El-jardali 2017 [55]	Lebanon	Generics	Pharmacist	153	Mixed‐model qualitative and quantitative research methods	Self-administered questionnaires and semi Structured Interviews	Medium
Emeka 2017 [56]	Saudi Arabia	Generics	Physician and pharmacist	58	Quant cross-sectional study	Face-to-face questionnaire	Medium
Fahmi 2022 [57]	Iraq	Biosimilars	Pharmacist	25	Mixed‐model approach combining qualitative and quantitative research methods	(1) Face-to-face or phone semi-structured interviews (2) Quantitative (ADR reports)	Medium
Fahmi 2022 [58]	Iraqi	Biosimilars	Physician	36	Qual interview	Semi-structured interviews face-to-face and virtual via Zoom	Medium
Farhat 2016 [59]	Arab countries, Iran,	Biosimilars	Physician and pharmacist	117	Quant cross-sectional study	Self-administered questionnaire	Medium
Gürler 2022 [60]	Turkey	Biosimilars	Physician	111	Quant prospective descriptive survey,	Web‐based questionnaire	Medium
Hadoussa 2020 [61]	Tunisia	Biosimilars	Physician	107	A quant descriptive and prospective study	Questionnaire via mail, face to face or phone interview	Medium
Hatem 2022 [62]	Lebanon	Generics	Physician	385	Quant cross-sectional descriptive study	Face‐to‐face interview using a questionnaire	Medium
Hatem 2023 [63]	Lebanon	Generics	Physician	270	Quant observational cross-sectional study	Face-to-face interview using a questionnaire	High
Mahdi 2020 [64]	Iraq	Generics	Physician	124	Quant cross-sectional study	Self-administered questionnaire	Medium
Mahmoud Soliman 2022 [14]	Egypt	Generics	Physician	100	Quant A retrospective chart review and survey	Self-administered questionnaire	Low
Mansour 2023 [65]	Lebanon	Generics	Pharmacist	80	A quant descriptive cross-sectional study	Self-administered questionnaire	Medium
Mohammed 2021 [66]	Iraq	Biosimilars	Pharmacist	264	Quant cross-sectional study	Web-based, self-administered questionnaire	Medium
Oncu 2021[67]	Turkey	Generics	Physician	305	A quant descriptive and cross-sectional study	Face-to-face interview using a questionnaire	Medium
Oqal 2022 [68]	Jordan	Biosimilars	Pharmacist	502	Quant A cross-sectional study	Web-based, self-administered questionnaire	High
Saleh 2017 [69]	Lebanon	Generics	Pharmacist and patient	25 pharmacists	Quant cross-sectional study	Self-administered questionnaire	Medium
Salhia 2015 [70]	Saudi Arabia	Generics	Physician	178	Quant cross-sectional study	Self-administered questionnaire	Low
Shraim 2017 [71]	Palestine	Generics	Pharmacist	302	Quant cross-sectional design	Self-administered questionnaire	Medium
Toklu 2012 [72]	Turkey	Generics	Physician and pharmacist	56 prescribers, 68 pharmacists	Quant cross-sectional study	Face-to-face questionnaire	Medium
Yousefi 2015 [73]	Iran	Generics	Pharmacist	1215	A quant descriptive cross-sectional study	Self-administered postal questionnaires	High
Yousefi 2015 [74]	Iran	Generics	Physician	410	A quant descriptive cross-sectional study	Self-administered questionnaire	Medium

### Data extraction and quality appraisal

Data extraction was performed independently by two reviewers (K.Q. and S.E.), and disagreements were resolved through discussion. A modified version of the Cochrane Effective Practice and Organization of Care Review Group (EPOC) data-collection tool was used to ensure systematic data extraction. To ensure usability, the extraction form was pilot-tested on ten randomly selected studies in Covidence before full implementation. However, manual extraction using a spreadsheet is preferred owing to its enhanced flexibility.

The extracted data included (i) study characteristics (country, population, setting, design, and participant count); (ii) data collection methods (self-administered structured questionnaires, focus groups, and semi-structured telephone and face-to-face interviews); and (iii) facilitators and barriers identified by physicians and pharmacists in the implementation of generic drugs and biosimilar policies.

Quality appraisals were conducted by two reviewers (K.Q. and S.E.) using the mixed methods appraisal tool (MMAT) version 2018 criteria [37]. The tool is designed for the systematic appraisal of the quality of various types of studies, including qualitative, quantitative, and mixed-methods research. It is useful to assess the methodological aspects of studies to determine their appropriateness, coherence, and rigor. No study was excluded based on the quality rating. Disagreements regarding the data extraction and quality appraisal results were resolved through discussion. The authors of the included studies were contacted to obtain additional information.

### Data analysis

Owing to the heterogeneity across study outcomes, data were analyzed via textual descriptions, groupings, clusters, and tabulations. The findings were synthesized based on the outcomes. The characteristics of the included studies were presented in a narrative format, as recommended by PRISMA.

## Results

### Study characteristics

**Fig. 1:**
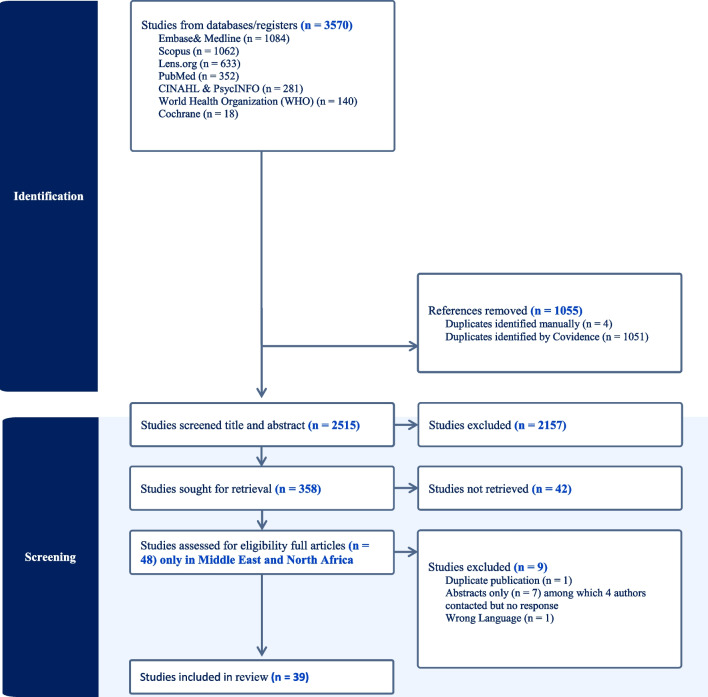
PRISMA shows the flow of identified studies through the review process

Of the 3570 citations reviewed, 39 studies targeting pharmacists and physicians were included (Fig. 1). These studies were published between 2012 and 2023 and used a range of methods. The study was conducted in Saudi Arabia (*n* = 9), Lebanon (*n* = 7), Iraq (*n* = 5), Jordan (*n* = 4), Turkey (*n* = 4), Iran (*n* = 3), Tunisia (*n* = 2), Egypt, Oman, Palestine, Qatar, and the United Arab Emirates. The characteristics of the included studies are shown in table (Table 1).

### Quality assessment

The quality of included studies varied, with some demonstrating strong methodological rigor and others demonstrating limitations in the study design. High-quality studies primarily utilize validated questionnaires and robust study designs to ensure reliable and well-supported findings. Three low-quality studies were identified, and two quantitative studies had weaknesses in sampling representativeness, validity of the measurement tools, and an unclear response rate. The qualitative study had some gaps, particularly in the analysis and interpretation of qualitative data and in detailing the role and potential biases of the researchers. These factors may have affected the robustness, generalizability, and validity of the findings.

The decision to include low-quality studies in this review ensured a comprehensive assessment of the adoption of generics and biosimilars in the MENA region, particularly in understudied countries, where research is scarce. Excluding these studies would risk publication bias, as high-quality research is often conducted in resource-rich settings, leaving gaps in evidence from less-developed healthcare systems. Despite methodological limitations, low-quality studies provide valuable contextual insights, allowing triangulation with high-quality research to identify consistent themes. We incorporated these studies to ensure a thorough representation of the findings, ensuring that policy recommendations are informed by the full spectrum of the available evidence.

### Facilitators and barriers addressing generic and biosimilars medicines identified by physicians and pharmacists

Of the 39 studies, 25 described the facilitators and barriers to generic medicine adoption by healthcare ecosystems identified by physicians and pharmacists**.** Facilitators included (i) availability of specific government policies promoting the use of generic drugs, (ii) understanding generics as an economic incentive for cost saving, (iii) awareness and education, (iv) availability of generic medicines, and (v) pharmacist empowerment and support for substitution (see Table 2). The barriers were as follows: (i) knowledge gap leading to a lack of trust in efficacy and safety; (ii) influence of pharmaceutical companies; (iii) cultural and psychological factors that create biases towards international brands; (iv) regulatory and policy implementation gaps; (v) communication difficulties and resistance; (vi) low consumer knowledge and awareness; and (vii) profitability concerns (Table 2).

**Table 2:** Facilitators and barriers addressing generic medicines identified by physicians and pharmacists

Authors, date	Population	Facilitators					Barriers						
		Availability of specific government policies promoting the use of generic drugs	Understanding generics as an economic incentive for cost-saving	Awareness and education	Availability of generic medicines	Pharmacist empowerment and support for substitution	Knowledge gap leading to lack of trust in efficacy and safety	Influence of pharmaceutical companies	Cultural and psychological factors	Regulatory and policy implementation gaps	Communication difficulties and resistance	Low consumer knowledge and awareness	Profitability concerns
Abduelkarem 2019	Physician and patient	✓	✓	✓			✓	✓	✓	✓	✓		
Abdulah 2019	Physician	✓	✓	✓			✓	✓	✓	✓			
Albadr 2015	Pharmacist		✓		✓				✓			✓	✓
Alkhuzaee 2016	Pharmacist	✓	✓	✓		✓	✓	✓	✓		✓	✓	
Al-Saadi 2023	Pharmacist			✓		✓	✓	✓		✓		✓	
Alsufyani 2023	Pharmacist	✓	✓			✓	✓				✓	✓	
Awada 2023 generic and biosimilar	Pharmacist			✓		✓	✓		✓				
Awaisu 2014	Pharmacist	✓	✓	✓		✓	✓			✓			
Baazaoui 2023	Physician	✓	✓				✓		✓	✓			
El-Dahiyat 2013	Pharmacist		✓	✓		✓	✓			✓	✓		✓
El-Dahiyat 2014	Physician		✓	✓						✓	✓		
El-Jardali 2017	Pharmacist			✓		✓				✓	✓		✓
Emeka 2017	Physician and pharmacist	✓	✓	✓					✓		✓		
Hatem 2022	Physician	✓	✓	✓			✓	✓			✓		
Hatem 2023	Physician and patient	✓	✓				✓			✓		✓	
Mahdi 2020	Physician	✓	✓	✓			✓		✓	✓			
MahmoudSoliman 2022	Physician		✓				✓				✓		
Mansour 2023	Pharmacist		✓	✓		✓	✓			✓			
Oncu 2021	Physician		✓	✓			✓		✓			✓	
Saleh 2017	Pharmacist and patient	✓	✓						✓		✓	✓	
Salhia 2015	Physician			✓			✓						
Shraim 2017	Pharmacist		✓	✓		✓	✓	✓		✓		✓	
Toklu 2012	Physician and pharmacist		✓				✓	✓			✓	✓	
Yousefi 2015	Pharmacist					✓	✓			✓			
Yousefi 2015	Physician	✓	✓				✓	✓			✓	✓	

Additionally, 15 studies acknowledged facilitators for biosimilars by physicians and pharmacists, including (i) biosimilar awareness, (ii) recognition of access benefits, (iii) perception of biosimilars as cost-effective alternatives, (iv) existing regulatory frameworks (FDA, EMA, and local), and (v) pharmacists’ professional roles and influence. Barriers to biosimilar adoption included (i) knowledge gaps and low familiarity with the products, (ii) inadequate long-term data, (iii) regulatory gaps, (iv) concerns about non-medical switching (NMS), (v) educational needs, (vi) nocebo effect concerns, (vii) supply and logistics issues, and (viii) cultural resistance (Table 3).

**Table 3:** Facilitators and barriers addressing biosimilars medicines identified by physicians and pharmacists

Authors, date	Population	Facilitators					Barriers							
		Biosimilars awareness	Access benefits recognition	Looking at biosimilars as a cost-effective alternative	Existing regulatory framework, FDA, EMA, local	Pharmacists’ professional role and influence	Knowledge gaps and low familiarity with the products	Inadequate long-term data	Regulations gaps	Concerns with non-medical switching (NMS)	Educational needs	Nocebo effect concerns	Supply and logistics	Cultural resistance
Abusara 2023	Pharmacist	✓	✓	✓			✓							
Almadany 2022	Physician		✓	✓				✓	✓	✓	✓			
Almalki 2020	Physician	✓			✓		✓				✓			
Almalki 2020	Pharmacist	✓		✓	✓		✓				✓			
Awada 2023	Pharmacist	✓					✓		✓		✓			✓
Chahine 2023	Pharmacist			✓		✓	✓		✓		✓			
Demirkan 2022	Physician	✓	✓					✓		✓		✓		
Fahmi 2022	Pharmacist			✓	✓			✓	✓	✓			✓	
Fahmi 2022	Physician			✓	✓		✓	✓		✓			✓	
Farhat 2016	Physician and pharmacist			✓	✓		✓		✓					
Gürler 2022	Physician			✓	✓		✓		✓					✓
Hadoussa 2020	Physician		✓	✓			✓		✓					
Mohammed 2021	Pharmacist			✓			✓		✓			✓		
Oqal 2022	Pharmacist			✓	✓		✓				✓			
Alahmari 2021	Pharmacist	✓		✓		✓	✓						✓	

### Facilitators addressing generic medicines

Of the 25 studies, 20 qualitative and quantitative studies highlighted the role of generics in reducing healthcare costs. Generic prescriptions serve as a strong cost-saving measure that significantly lowers healthcare expenditure. Both physicians and pharmacists recognized these economic benefits as key motivators. Sixteen studies emphasized the importance of education and awareness in promoting generic drug use. Physicians and pharmacists with knowledge of “generic” and “bioequivalence” demonstrated more positive perceptions and greater policy support. Enhancing our understanding of the efficacy and safety of generic drugs among healthcare providers and patients is crucial for their acceptance and utilization. Educational interventions improve prescribing behaviors, particularly when integrated into academic curricula and professional development programs.

Twelve studies cited the need for government policies to support the use of generic drugs. This includes regulatory requirements for bioequivalence data before market approval, policies promoting generics, and regulations that allow pharmacists to substitute generics. Ten studies identified a strong link between pharmacist empowerment and generic substitution, facilitating the broader acceptance of generics. Policies enabling pharmacists to substitute generics enhance their role in patient education and medication selection, optimize healthcare costs, and improve access. Physicians also recognize pharmacist empowerment as a means of improving healthcare-spending efficiency. Although only one study identified the availability of generic medicines as facilitators, ensuring access to these medications remains a crucial factor [75].

### Facilitators addressing biosimilar medicines

Twelve studies agreed that biosimilars offer a cost-effective solution for reducing healthcare costs. Their affordability is particularly valuable in healthcare systems, burdened by the high cost of biological medicines, making financial benefits a major driver of biosimilar adoption. Seven of the 15 studies highlighted the role of regulatory frameworks (FDA, EMA, and local authorities) in supporting the use of biosimilars. For instance, guidelines from the Saudi Food and Drug Authority (SFDA) assist physicians in making informed decisions [3, 76]. Similarly, regulatory endorsements from bodies such as the FDA and EMA are major facilitators in multiple Arab countries, including Iran and Turkey [77, 78].

Six studies reported that awareness enhanced the acceptance of biosimilars in clinical settings. Four studies emphasized the role of biosimilars in improving healthcare accessibility. For example, in Saudi Arabia, rheumatologists have noted that biosimilars have expanded patient access to biological treatments for rheumatic diseases [79]. Two studies highlighted the impact of pharmacist empowerment on biosimilar integration. In Lebanon, pharmacists see themselves as key stakeholders in promoting biosimilars [2]. In Saudi Arabia, pharmacists play a crucial role in introducing and managing biosimilars, educating patients and healthcare professionals, and ensuring their proper use [75].

### Barriers addressing generic medicines

Twenty of the 25 studies highlighted gaps in knowledge about generic manufacturing standards, leading to distrust in efficacy and safety. These gaps, particularly among physicians, limit the use of generic prescriptions. In addition, deficiencies in understanding and communicating bioequivalence and therapeutic equivalence in patients must be addressed. Thirteen studies have identified gaps in the enforcement of regulatory policies. For instance, Jordanian legislation restricts pharmacists from substituting generics without prescribing approval, thus limiting their autonomy [4]. In the UAE, while policies promote generics, the exclusion of narrow therapeutic index drugs from substitution policies reduces their overall impact [13].

Twelve studies identified communication barriers between pharmacists, prescribers, and patients, making generic substitution challenging. In Saudi Arabia, over half of pharmacists do not consult physicians before substitution, owing to time constraints or perceived irrelevance [5]. Patient resistance, often driven by quality concerns, further complicates adoption. In Lebanon, physicians frequently overuse the “non-substitutable” option, restricting their ability to offer generics [6]. Some pharmacists have reported that physicians discourage patients from accepting substitutions, thus undermining their generic substitution [4]. Ten studies cited low consumer awareness as a major barrier, leading to reluctance to accept generic medications and a preference for branded products. Ten studies noted biases favoring international brands over local brands. Physicians and patients often perceive multinational company medications as superior in quality and reliability. This preference may stem from cultural biases or the perceived prestige of brand-name drugs.

Eight studies raised concerns about pharmaceutical companies promoting more expensive brand-name drugs. Physicians and pharmacists acknowledge that industry-sponsored activities, including medical samples and conference funding, could bias prescription habits. Only three studies raised the issues of profitability and gains. In Lebanon, over half of the pharmacists felt that the pricing system discouraged generic substitution, as branded drugs often yield higher profit margins, thus disincentivizing the promotion and use of cheaper generic alternatives by community pharmacists [6].

### Barriers addressing biosimilar medicines

Twelve studies identified knowledge gaps regarding biosimilar interchangeability, efficacy, and safety that hindered their adoption. Misconceptions regarding biological drug variability, approval processes, and biosimilar costs contributed to hesitation in recommending biosimilars to patients and other healthcare providers. In Jordan, pharmacists reported difficulty recognizing biosimilar brand names, affecting appropriate dispensing and counseling [80]. Physicians in Iraq and Tunisia were cautious about prescribing biosimilars, particularly in sensitive patient populations such as oncology and hematology [58, 61].

Seven studies cited the absence of clear regional regulatory guidelines as the major barrier. Unclear regulations on interchangeability and automatic substitution hinder pharmacists’ autonomy. In Lebanon, pharmacists are not allowed to substitute biosimilars without prescriber consent, which slows their adoption [2]. Six studies highlighted the lack of comprehensive educational programs on biosimilars. Physicians and pharmacists expressed the need for structured training on biosimilar development, regulatory pathways, clinical use, safety, and efficacy. Addressing this gap through university curricula and continuous professional development can improve the confidence in biosimilars.

Physicians in six studies were hesitant about NMS, particularly rheumatologists in Turkey and Saudi Arabia. Concerns about potential clinical differences and health impacts discourage switching stable patients from originator biologics to biosimilars [79, 81]. Four studies noted that the limited long-term data and clinical experience hindered the adoption of biosimilars. In Turkey, pediatric rheumatologists cited the lack of clinical trials and real-world data as a key barrier, with 64% expressing concerns about safety and efficacy in children [81]. Two studies reported the nocebo effect, in which negative patient expectations can influence patient and caregiver perceptions and adherence to biosimilar treatments [14, 81].

Three studies highlighted inconsistent biosimilar supply chains and logistical hurdles affecting market penetration. In Iraq, unreliable supply chains and regulatory barriers have been noted as significant challenges [58, 82]. Two reports have identified cultural resistance as a barrier. Some physicians hesitate to adopt biosimilars, especially for long-term treatment, owing to concerns about interchangeability and substitution protocols. This reluctance is more pronounced in regions where biosimilars are relatively new [78, 83].

### Pharmacists and physicians comparative views

An analysis of 39 studies highlighted distinct differences in how physicians and pharmacists perceive and integrate generics and biosimilars into healthcare systems. A direct comparison of perceived facilitators and barriers was made and quantified through the proportion of studies that addressed distinct aspects among the respective groups (Table 4).

**Table 4:** Frequency and proportion of studies addressing specific views on the adoption of generics and biosimilars among pharmacists and physicians

Aspect	Biosimilars (physicians) (n* = 7)	Biosimilars (pharmacists) (*n* = 9)	Generics (physicians) (*n* = 12)	Generics (pharmacists) (*n* = 14)
Economic benefit recognition as facilitator	71% (5)	89% (8)	100% (12)	71% (10)
Regulatory framework support as facilitator	57% (4)	44% (4)	67% (8)	36% (5)
Awareness and education need as facilitator	29% (2)	44% (4)	67% (8)	64% (9)
Pharmacist’s autonomy in substitution as facilitator	0%	22% (2)	8% (1)	64% (9)
Knowledge gaps as barrier	71% (5)	89% (8)	92% (11)	71% (10)
Cultural resistance as barrier	14% (1)	11% (1)	50% (6)	36% (5)
Non-medical switching (NMS) concerns as barrier	57% (4)	22% (2)	N/A	N/A
Influence of pharmaceutical companies as barrier	N/A	N/A	42% (5)	29% (4)
Low consumer knowledge and awareness as barrier	N/A	N/A	33% (4)	50% (7)

*Number of studies

Both physicians and pharmacists overwhelmingly supported generics and recognized them as well-regulated and cost-effective alternatives. Regarding biosimilars, 89% of pharmacist-led studies documented pharmacists perceiving these products as cost-effective alternatives, in contrast to 71% of studies involving physicians. Pharmacists play a leading role in dispensing generics and supporting substitution policies, contrary to biosimilars, where although pharmacists had a more positive outlook, they lacked decision-making power. Unlike generics, biosimilars require prescriber approval before substitution, which limits their adoption autonomy. Only two studies identified pharmacists as key influencers in biosimilar adoption, indicating that pharmacists play a limited role in biosimilar adoption owing to strict regulatory substitution controls.

Similar barriers for both generics and biosimilars included knowledge gaps (67% for physicians and 64% for pharmacists), regulatory hurdles (50% for both), and cultural resistance (67% for physicians and 36% for pharmacists). For biosimilars specifically, hesitancy in switching stable patients remains high among physicians compared to pharmacists owing to immunogenicity concerns and uncertainty about treatment variations. The nocebo effect has been reported only in physician-based studies, highlighting its influence on patient perceptions. This also pinpoints the reference to awareness and education as important facilitators to bridge the knowledge gaps that will help boost physician confidence in biosimilars and enhance pharmacist-led substitution policies.

For generics, the influence of pharmaceutical companies and promotional activities influenced physicians slightly more than pharmacists and could bias prescription habits towards brands. Additionally, studies noted slightly more preference for multinational brands over generic brands in the physician’s population, leading to some hesitancy in prescription due to cultural resistance. Low consumer knowledge and awareness negatively affected the extent of pharmacists’ adoption of generics, and customer resistance affected their dispensing decisions.

## Discussion

This study identified the facilitators and barriers identified by physicians and pharmacists concerning generic and biosimilar products in the MENA region. Perceptions, attitudes, and views were explored with the intention of using these findings to determine approaches that could be used by governments and healthcare payer organizations to improve the acceptance of these cost-saving alternatives and ultimately facilitate patients’ access to essential and life-changing medications. All 39 included studies had been published since 2012, addressing generics and biosimilar drugs, suggesting a growing interest regionally in these products.

While both generics and biosimilars aim to provide cost-effective alternatives to brand name therapies, the facilitators of their adoption differ owing to variations in development complexity, regulatory requirements, market dynamics, and policies governing their interchangeability. In terms of complexity and development, generic drugs are small-molecule compounds synthesized through straightforward chemical processes, resulting in identical replicas of brand-name drugs. This simplicity facilitates easier manufacturing and quality control, contributing to widespread infiltration. In contrast, biosimilars are large, complex molecules derived from living organisms, making their development and production more intricate [84]. The approval process for generic drugs involves demonstrating bioequivalence with the original drug, allowing for a streamlined pathway that bypasses extensive clinical trials. This accelerated entry into the market. However, biosimilars must undergo comprehensive evaluations, including analytical studies and clinical trials, to establish similarities to the reference biologic, without significant clinical differences. This rigorous process ensures safety and efficacy, but can prolong the time to market [85]. With regard to market dynamics and economic incentives, the generic drug market is characterized by high competition, with multiple manufacturers producing the same medication, leading to significant price reductions and widespread adoption. However, biosimilars face a less saturated market owing to the complexities and costs associated with their development and manufacturing. Consequently, although biosimilars offer economic incentives, they may not be as effective as those for generics [86]. Furthermore, generics are often deemed interchangeable with their brand name counterparts, allowing pharmacists to substitute them without prior physician approval and facilitating easier integration into healthcare systems. However, biosimilars require additional regulatory approval to be considered interchangeable, meaning that substitution decisions often remain under the purview of prescribing physicians, potentially slowing adoption rates [87].

Five facilitators were identified by physicians and pharmacists for each generic and biosimilar drug, while there were seven barriers to generic utilization and eight barriers to biosimilars. Our findings led us to make the following observations: common facilitators and barriers for physicians and pharmacists to accept generics and biosimilars in their practice in the MENA region were similar to those previously identified globally [33, 88].

The main barrier is the knowledge gap among physicians and pharmacists, which is often related to product safety and efficacy. This has also been identified as a barrier in European countries, affecting trust and willingness to use products [89]. Facilitators, such as understanding the role of products in healthcare systems cost saving, the opportunity to grant access to a broader population, and frameworks and regulations that govern the generics and biosimilar market, were the main facilitators of physicians and pharmacists. Policies and guidelines are the most cited group of enablers, as identified by Rieger et al. in their recent publication, to encourage the uptake of biosimilar medications [90]. Pharmacists’ empowerment and support for substitution and their professional role and influence were only seen at the pharmacist level, although their role is well recognized among healthcare ecosystem stakeholders, especially the role of clinical pharmacists in the utilization of biosimilars, where they act as educators to drive rational drug use [91]. A role that is missing and needs to be addressed in the MENA region, where pharmacists, if allowed to substitute biosimilars, can play a driving role in active uptake.

The adoption of generic medicines and biosimilars in the MENA region is influenced by distinct barriers, each shaped by the unique factors inherent in these pharmaceutical categories. Barriers specific to generic medicines include the influence of pharmaceutical companies, in which brand-name pharmaceutical companies often engage in aggressive marketing strategies and offer financial incentives to healthcare providers to favor their products over generics. This practice can lead to a preference for branded medications among physicians and pharmacists, thereby hindering the adoption of generic alternatives [6]. A significant barrier to generic medicine adoption is the lack of consumer awareness of the efficacy and safety of generic medicines. Many patients harbor misconceptions, believing that brand name drugs are superior, which leads to resistance against substituting prescribed medications with generic versions [92]. Sometimes, pharmacists may be reluctant to dispense generic medicines because of their lower profit margins compared to brand-name drugs. This economic consideration can discourage pharmacists from promoting or substituting generic drugs, thereby affecting their availability to patients [6]. However, barriers specific to biosimilars include their relative novelty, which means that long-term safety and efficacy data are limited. This paucity of comprehensive longitudinal studies leads to caution among healthcare providers when considering biosimilars as treatment options [90]. Physicians express apprehension about switching patients from original biologic therapies to biosimilars for non-medical reasons such as cost savings. The concern focuses on potential variations in clinical outcomes, which may affect patient health [8], and the nocebo effect, where negative expectations about a treatment lead to poorer health outcomes, is a notable concern with biosimilars. Patients’ skepticism or negative perceptions of the efficacy of biosimilars can adversely influence treatment effectiveness, posing a barrier to their adoption [8]. Both generics and biosimilars face common challenges in the MENA region, including cultural and psychological biases favoring international or brand-name products over locally produced or generic alternatives. This cultural inclination affects both patient acceptance and physician prescription habits, thereby impeding the adoption of generics and biosimilars [93, 94]. Effective collaboration between physicians and pharmacists is crucial for successful integration of generic drugs and biosimilars into treatment protocols. However, communication barriers and lack of mutual recognition of professional roles can hinder this partnership, affecting medication substitution practices [95, 96].

Facilitators of the international adoption of generics and biosimilars have been advocated to alleviate the financial burden of escalating drug costs. Different healthcare systems have adopted various policies to encourage the adoption of generic drugs and biosimilars. Established guidelines from reputable regulatory bodies such as the FDA and EMA provide a framework that supports the adoption of generics and biosimilars by ensuring their safety and efficacy [97]. Other policy examples include setting price caps, allowing or mandating drug substitution, and using reference-pricing strategies. Interventions in Europe under the “4 Es” (education, engineering, economics, and enforcement) has successfully promoted the use of generics with the aid of the pricing policies that cap generics prices at 20–50% of their branded equivalents [98]. At the healthcare provider and organization levels, some countries have set prescription quotas as incentives [99], whereas others have adopted profit-sharing models [100], especially in biosimilars [101]. Pharmacists helped in promoting biosimilar adoption through legally acceptable substitution policies in France and the USA [102, 103]. The Australian government has implemented measures to increase biosimilar uptake by promoting their prescription to treatment-naïve patients and streamlining the approval process for these drugs [104]. All of these initiatives emerged from the slow adoption of these cheaper versions of medication.

A significant barrier is the limited knowledge of health care providers regarding biosimilars. This lack of familiarity can lead to reluctance to prescribe these medications [105]. The high cost of developing and commercializing biosimilars can deter manufacturers, thereby affecting market availability. Additionally, market conditions and regulatory protection pose challenges for the growth of biosimilar markets [106]. Educational strategies are essential to overcome these barriers and promote the adoption of generic and biosimilar medicines among healthcare professionals. The implementation of comprehensive educational programs enhances our understanding of the safety, efficacy, and regulatory pathways of these medicines. Addressing misconceptions and building confidence is a crucial component of education. The FDA’s Biosimilars Action Plan includes developing educational resources to dispel myths and misconceptions regarding biosimilars to enhance acceptance among healthcare providers and patients [107]. Engaging multiple stakeholders, including health care professionals, patients, policymakers, and the public, fosters a more accepting environment for biosimilars. For example, Germany’s substantial commitment to educating physicians and the European Specialist Nurses Organization’s (ESNO) development of targeted educational programs for nurses are aimed at facilitating patient transitions to biosimilars [108].

Policy and regulatory support also play a significant role in promoting generic and biosimilar adoption. By implementing these strategies, healthcare systems can enhance the acceptance and utilization of generic and biosimilar medicines, ultimately improving patient access to affordable treatment [104]. Meanwhile, the educational need to bridge the knowledge gaps for both generics and biosimilars has been highlighted as a major concern and obstacle for physicians and pharmacists in the Middle East and internationally [108, 109]. Strategies to enhance acceptance of generics and biosimilars in the healthcare ecosystems of the MENA region should focus on the need for comprehensive educational programs. Despite existing resources, there is still a significant need for comprehensive educational programs, either at the university level or as a continuous professional development course. These courses should be tailored to generic manufacturing standards, concepts of bioequivalence, and therapeutic equivalence, to guarantee the efficacy and safety of generics. Moreover, programs should address the biosimilar development process, regulatory pathways, clinical use, and variability of biological drug formulation, safety, and efficacy. All these critical elements should be clear to both physicians and pharmacists to better understand the basic concepts and ultimately pave the way to guide healthcare providers to build trust in efficacy and safety, which currently hinders their preference for generics and biosimilars over brand-name medications.

Interestingly, most of the included studies were conducted in Saudi Arabia, Lebanon, and Iraq, with only one being conducted in Egypt and the UAE, indicating the need to explore this situation in these countries. Generics and biosimilars have been approved for many years; however, their acceptance and maximum utilization have not yet been achieved in this region. It appears that the use of these cost-containment measures is still immature and needs to be tailored to regional needs and culture, so they will be acceptable among all pillars of the MENA healthcare ecosystem.

### Strengths

This systematic review is a pioneering effort to identify the facilitators and barriers encountered by physicians and pharmacists while implementing generic drug and biosimilar policies within the Middle East and North Africa (MENA) region. By synthesizing diverse evidence, this study offers valuable insights into the regional acceptance of pharmaceutical products. This comprehensive approach ensures a nuanced understanding of the factors influencing policy implementation in various healthcare settings.

### Limitations

A notable limitation of this review is the inaccessibility of four pertinent publications despite attempts to contact the authors via email [110–113]. The titles of these articles suggest that they assess physicians’ or pharmacists’ knowledge, attitudes, or perceptions of generics or biosimilars, indicating that their exclusion may result in an incomplete analysis. Additionally, the included studies utilized varied measures to identify facilitators and barriers, which, while appropriately synthesized, could introduce heterogeneity, affecting the consistency of the findings. Furthermore, the timeframes of the included studies may not accurately reflect current scenarios, as older studies might not capture recent developments in the field.

## Conclusion

In the Middle East and North Africa (MENA) region, the adoption of generic and biosimilar medications was shaped by various facilitators and barriers identified across 39 studies. Supportive government policies, recognized cost savings, and the empowerment of pharmacists have been instrumental in promoting the integration of generics into healthcare systems. However, significant challenges persist, including knowledge gaps, regulatory hurdles, cultural resistance, and patient misconception. Similarly, although biosimilars offer cost-effective alternatives to biological originators, their widespread adoption is hindered by regulatory, clinical, and market-related challenges. Physicians tended to be more receptive to prescribing generics than biosimilars; however, both categories encountered trust-related obstacles. Pharmacists generally support the use of both generics and biosimilars but often face regulatory constraints that limit their ability to substitute biosimilar medications. While the economic benefits of these medicines are widely acknowledged, concerns regarding their safety, substitution policies, and non-medical switching (NMS) impede their broader acceptance. Physicians played a pivotal role in the adoption of biosimilars, whereas pharmacists played a key role in facilitating generic substitution. Addressing these challenges through targeted educational initiatives, harmonized regulations, and policies that empower pharmacists is essential. Strategic initiatives at the regulatory and payer levels, including education, can raise awareness and build trust, ultimately fostering a culture that embraces generics and biosimilars in the MENA healthcare ecosystems. Such strategies can enhance the integration of generics and biosimilars into MENA healthcare systems, ultimately improving patient access to affordable treatments.

## Supplementary Information

Below is the link to the electronic supplementary material.

Supplementary file1 (DOCX 21 KB)

## Data Availability

No datasets were generated or analysed during the current study.
