# Supplementary material for: Facilitators and barriers to generic and biosimilar medications in the Middle East and North Africa: insights from physicians and pharmacists—a systematic review
Source: Eur J Clin Pharmacol. 2025 Mar 14;81(5):647–65. doi: 10.1007/s00228-025-03819-5 (PMC12003508; doi:10.1007/s00228-025-03819-5)
Supplement: Supplementary file 1 — Supplementary file1 (DOCX 21 KB) [file 228_2025_3819_MOESM1_ESM.docx]

**Fig. 1 Ovid MEDLINE and EMBASE** **databases search strategy**

Embase <1974 to 2024 February 26>

Ovid MEDLINE(R) ALL <1946 to February 26, 2024>

1 pharmacist*.mp. or pharmacist/ 179155

2 physician/ or physician*.mp. 1528220

3 general practitioner/ or General Practitioner*.mp. 215122

4 Medical Practitioner*.mp. 17426

5 clinician/ or clinician*.mp. 779468

6 health care personnel/ or healthcare provider*.mp. 311922

7 Healthcare Professional*.mp. 103460

8 doctor*.mp. 491905

9 1 or 2 or 3 or 4 or 5 or 6 or 7 or 8 3079325

10 Drug*, Generic. mp. or generic drug/ 20568

11 (generic drug* or generic medication* or generic medicines*).mp. 20004

12 Biosimilar Pharmaceutical*.mp. or biosimilar agent/ 11001

13 Biological Product*.mp. or biological product/ 104761

14 biosimilar*.mp. 18419

15 10 or 11 or 12 or 12 or 14 41422

16 advantage*.mp. 1225668

17 benefit*.mp. 2448544

18 challenge*.mp. 2163129

19 barrier*.mp. 1016089

20 Perception*.mp. or perception/ 1096053

21 physician attitude/ or pharmacist attitude/ or attitude/ or Attitude. mp. or health personnel attitude/ 871135

22 Health Knowledge. mp. or attitude to health/ 346263

23 personal experience/ or experience*.mp. or work experience/ 3431278

24 facilitator*.mp. 93578

25 opinion*.mp. 344897

26 view*.mp. 1293966

27 behavior/ or behaviours*.mp. 348761

28 16 or 17 or 18 or 19 or 20 or 21 or 22 or 23 or 24 or 25 or 26 or 27 11880834

29 systematic review. mp. or "systematic review"/ 900125

30 meta-analysis/ or meta-analys*.mp. 797658

31 observational study. mp. or observational study/ 619682

32 qualitative research/ or Qualitative Research. mp. 245895

33 Qualitative study. mp. 143052

34 video interview/ or interview/ or semi structured interview/ or telephone interview/ or interview*.mp. 1168411

35 questionnaire. mp. or questionnaire/ or Health Perceptions Questionnaire/ 2160119

36 cross-sectional study/ or Cross-Sectional Stud*.mp. 1247616

37 Surve*.mp. or Surveys/ 4260110

38 Health Care Surveys. mp. or health care survey/ 59884

39 Health Surveys. mp. or health survey/ 315176

40 prospective study. mp. or prospective study/ 1701915

41 mixed method. mp. 23889

42 observational study. mp. or observational study/ 619682

43 focus group. mp. 82314

44 29 or 30 or 31 or 32 or 33 or 34 or 35 or 36 or 37 or 38 or 39 or 40 or 41 or 42 or 43 9216728

45 9 and 15 and 28 and 44 1800

46 45 and 2012:2024.(sa_year). 1416

47 limit 46 to the English language 1386

48 remove duplicates from 47 1084

**Table 1: Inclusion and exclusion criteria according to PICO framework**

| PICOs | **Inclusion criteria** | **Exclusion criteria** |
| --- | --- | --- |
| Population | Physicians and pharmacists working within healthcare systems in the MENA. | - Non-physician or non-pharmacist healthcare professionals. - Studies focus solely on patient perspectives without physician or pharmacist input. - Studies focus solely on payers or the pharmaceutical industry’s perspectives without physician or pharmacist input. - Physician or pharmacist students. |
| Intervention | Generic and biosimilar medicines policies implemented within healthcare systems. |  |
| Comparator | Not using generic and biosimilar medications. |  |
| Outcome | Primary outcomes   - Identification of advantages and challenges perceived by physicians and pharmacists. - An objective measure of the following should be addressed in the study to be included: - Perceptions/opinions/behaviors/views about generic and biosimilar medicines, presented as   - Quantitative data presented as the proportion of participants   holding perceptions about generic and biosimilar medicines.   - Best to include self-report measures to evaluate general perceptions about generic and biosimilar medicines, presented as percentages of the total sample assessed.   - Qualitative data | Studies that do not report outcomes relevant to the implementation of generic and biosimilar medicines policies. |
| Study design | - Primary research studies include:   - Observational studies   - cross-sectional studies   - prospective studies   - mixed methodology   - Qualitative studies   - Any other study methodology leading to a publication within the scope of this review. | - Editorial opinions, letters to the editor, and other ‘opinion ’-based publications. |
